# Inquiry into the Physical Constitution of Water, the Structure of Fluids, and the Cause of Fluidity in General

**Published:** 1813-01

**Authors:** Thomas Charlton Speer

**Affiliations:** President of the Royal Physical Society, Edinburgh


*For the Medical and Physical Journal.*

### INQUIRY *into the* PHYSICAL CONSTITUTION *of* WATER, *the* STRUCTURE *of* FLUIDS, *and the* CAUSE *of* FLUIDITY *in general.*

(Continued from Vol. 28, p. 438.)

I SHALL now more particularly consider them in their application to water itself.

*Water considered as a Fluid.*—Water may be said to be the most fluid of all fluid bodies, and indeed perhaps indispensable to the existence of fluidity*—1st, from the very slight intensity in that cohesive force by which its atoms are connected;

* By the word *fluids* I mean here, of course, non-elastic fluids; I omit all exceptions in the case of ether, alcohol, oils, &c.

connected: hence the increased interstice and increased capacity for caloric; 2dly, from the minuter size of the ultimate molecule of each particle; and 3dly, from their form being such as to be best adapted for motion, viz. globes rolling through each other.

Hence, in the gradation of the *density* of bodies, (by which I mean that power resulting from the union of attraction, of cohesion, and gravitation,) viz. of the solid, fluid, and aeriform states, water may be placed at the top of the fluids thus occupying the intermediate link between fluids and gases; this scale beginning with the densest solid, and ending with the rarest gas.

It was said by Sir Isaac Newton, and afterwards, I believe, by Boerhaave, that the fluidity of water was invariable; that its tenacity or viscousness, as they termed it, was the same at its passage to the boiling, as the freezing, point; and that the vibrations of a pendulum were as quick in the one as the other.

Although, at first view, this may appear perfectly irreconcileable with the known laws of attraction and caloric, and although the point has been much disputed and invalidated by the experiments of later philosophers, yet a little consideration may, I think, throw some light on this problem.

If a mass of fluid such as water consist of a series of spheres or globes rolling through each other, its mobility cannot much be altered so long as its particles retain this sphericity or globularity of figure; that is, so long as the mass remains between the limits of fluidity, say between 32° and 212°. If the mass be heated, the bulk will, of course, be increased; but why? merely because the interstices are increased. The mobility of the particles is in no wise affected, because their figure is not affected, and indeed herein consists the wide distinction between expansion and fluidity, or between *change of quantity* and *change of quality* in the cohesive force, as already so often alluded to. If the quality is altered, the figure of each atom must be altered; and, if the figures are altered, the tenacity or viscousness of the mass must be altered.

Before any thing conclusive can be advanced respecting the variations of fluidity in water, we must be in possession of more accurate trials than what have hitherto been made. The experiment of the pendulum seems liable to abundant sources of fallacy, as well arising from the nature and construction of the machine itself, as from those quick variations of temperature which must occur in the water. Perhaps a more satisfactory experiment would be that of comparing the differences of time between the ascents of two air bubbles bles in two very tall glass graduated jars, at different temperatures, say one at 50°, the other at 150°. Liquid water, possessing such perfect fluidity, and such perfect homogenity in its structure, its weight must, consequently, be more equable and independent; its constitution rendering it less subject to the adventitious influence of those circumstances by which the weights of other fluids become often so variable: hence it has become the standard by which the specific gravity of bodies is estimated. The body is first weighed in air, then in water; the loss of weight it acquires in the latter is equal to that of a mass of the fluid of the same dimensions as the solid—thus, the loss of difference is the divisor, the weight in air the dividend, and the specific gravity is the quotient.

*Water considered as a Solid.*—When water is reduced to 32°, its structure undergoes very remarkable alterations. The cohering lines or radii between the particles move or emanate with greater velocity; their direction is changed; their number is increased; they have less spaces to traverse; their power is left more to itself, and unshackled by any opposite one; the alternating spaces, which, according to Boscovich, occupy the interval between two atoms, no longer exist; the chain is gradually destroyed; the limits of repulsion grow narrow, those of attraction wide; the cohering lines, instead of being directed equally all around each atom with equal force, now move in one determinate line, and with different forces: hence the shape of each particle must alter and be dependant on the points of surface with which the cohering lines from its neighbouring atom come into contact. These points are much increased in number, because the lines are increased in number; thus new surfaces for approximation are exposed, interstices become blocked up, points of condensation soon originate in the mass, spicula and filaments of regular forms and angles soon arise; these go on increasing, and form nuclei for others to rest on, and thus the whole mass becomes solid by that process which we call *crystallization.*

From this process it would evidently appear that the mass of water must suffer great contraction in its dimensions: quite the contrary. In this case, water affords a most remarkable exception; when cooled to 40°, instead of continuing to contract, it expands down to 32°.

This very striking anomaly in the instance of water, has been variously accounted for. Mairan's explanation seems very just. He attributes it to a certain peculiar polarity in the particles of the water, by which they prefer particular sides of union; but why should water require so many de-

 grees grees for such polarity to take effect? We know that some saline solutions suffer a very slight expansion at the very instant of congelation; but water begins at eight degrees warmer than this point.

Perhaps the only way by which we can reconcile this, is by considering the very superior homogenity of the structure of water; the very superior uniformity of its particles in size, form, and distance from each other; the greater perfection with which their balance is adjusted; their greater insensibility to external impressions; and hence their greater liability to preserve this balance unaltered. From these considerations we may, perhaps, be allowed to conclude that a greater quantity, both of time and space, is necessary for the process of congelation.

Water then takes up more room in its solid than fluid state; and hence ice will float on liquid water, so that when set by to congeal in a close vessel, as much room should be left for this increased expansion as results from the difference of bulk in equal weights of ice and water. If this is not attended to, the most dreadful consequences will arise, as is well known;* the strongest vessels may be burst; and hence, in severe frosts, when the sap of trees congeal, they are torn asunder violently.

*Water considered as a Gas.*—On the other hand, when water ascends in the scale already mentioned, and that its temperature rises to 212, its general alteration of structure and physical appearance become no less remarkable than in its descent, viz. into solidity. However the specific transitions that induce this change are not of so decided and marked a character. I conceive a gas to be the same in structure, as to the shape of its atoms, with a liquid, and that the great difference may consist in a change of quantity, not a change of quality, in their cohesion, and hence in a change of interstice. This change of quantity must, however, be so very great, that the alternating spaces which Boscovich alludes to, can bear no proportion to each other. Those of repulsion must far exceed, and thus *elasticity*, a new power, must be brought into play. A mass of gas like a mass of liquid, may consist of rolling circles, spheres, or globes; their rotundity rendered still more perfect, and consequently their mobility; and the size of the ultimate molecule much reduced; and thus vaporization may only produce a change of weight, not a change of form.

---

* For a full and interesting account of effects of this kind, see Exper. de l'Acad. del Cimento (of Florence).

These

These two conditions then alone may be sufficient to constitute a gas, viz. change of interstice and change of size; whereas to constitute a solid, change of form, change of size in the atoms, and change of interstice between them, are necessary.

This is rendered still more probable, at least in the structure of water rendered gaseous, when we consider how susceptible it is of re-assuming its liquidity, when the balance of interstice is restored, and the great ease with which this can be done. For water to descend from the liquid to the solid states, a positive degree of cold is alone sufficient; to descend from the gaseous to the liquid states, it is influenced and assisted much by other circumstances, viz. *pressure.* The only effect of this can be to diminish interstice and produce contraction. Vaporization then does not solely depend on temperature, but on temperature modified by pressure.*

This effect of pressure on the vapor of water, and on the constitution of elastic fluids in general, is most powerful. It is known that the distances between the centres of their atoms is always inversely as the cube root of their density. Thus, if the density of a mass of gas be 1, and this mass be *pressed* into $\frac{1}{8}$ of its bulk, its density becomes 8: here then we have the distances inversely as the cube root of 1 to that of 8, or 1 : 2.

On the constitutions of liquid, water has no such effect. Various experiments have been tried, insomuch as to make the water exude through the pores of the vessel, but all to no purpose.†

The constitution of steam differs, however, from that of other gases, as the constitution of ice differs from that of other crystals. The particular transitions which occur in the production of steam, are well known. They of course much depend on pressure. On this principle M. Papin, Professor of Mathematics at Marburgh, invented his Digester in 1680. His intention was to dissolve bones: this it will do. The water being pressed and squeezed on all sides, seeks for refuge in the smallest interstice, and thus the bone is disintegrated. Water, it is said, may acquire a heat of 400° in it, and become red hot. The most remarkable property of steam is its *elasticity;* this may be said to constitute *the true vaporous principle.* According to M. Desa-

---

* For the very accurate and highly-ingenious observations and experiments of Mr. Dalton, and also Mr. W. Henry, on this subject, see Manchester Memoirs, vol. v.

† The experiments of Mr. Muschenbroke and M. Duhamel particularly shew this.

gulier it is 14,000 times more dilatable than water; so that, if a drop of water is introduced into a vessel whose dimensions in relation to the water are 1400 : 1, if the drop be vaporized, the vessel will be full.

Hence we see, in all the stages of water, that uniformity of influence which the attracting and repelling powers have over its atoms. As a solid, we see in it the effects of the former; as a gas, we see in it the effects of the latter; and, as a liquid, we see it balanced by both.

Thus then these three states of water, though so distinct from each other, and though separated in their properties by fixed and determinate limits, still, however, may be said to constitute one and the same chain, equally whole and complete in all its parts. As ice, it affords the most perfect process of crystallisation; as water, it possesses the truest character of fluidity; and, as steam, it possesses the most peculiar and distinguishing properties of an elastic fluid.

This chain, therefore, presents every modification to which the laws of material structure are subject; and hence the *physical constitution* of water affords the most perfect and legitimate result of that arrangement by which these laws are regulated.

THOMAS CHARLTON SPEER, M.D.
President of the Royal Physical Society, Edinburgh.

